# Supplementary material for: Evaluating the effects of embedded self-massage practice on strength performance: A randomized crossover pilot trial
Source: PLoS One. 2021 Mar 2;16(3):e0248031. doi: 10.1371/journal.pone.0248031 (PMC7924734; doi:10.1371/journal.pone.0248031)
Supplement: S2 File — CONSORT checklist (extension for pilot trials). (DOC) [file pone.0248031.s002.doc]

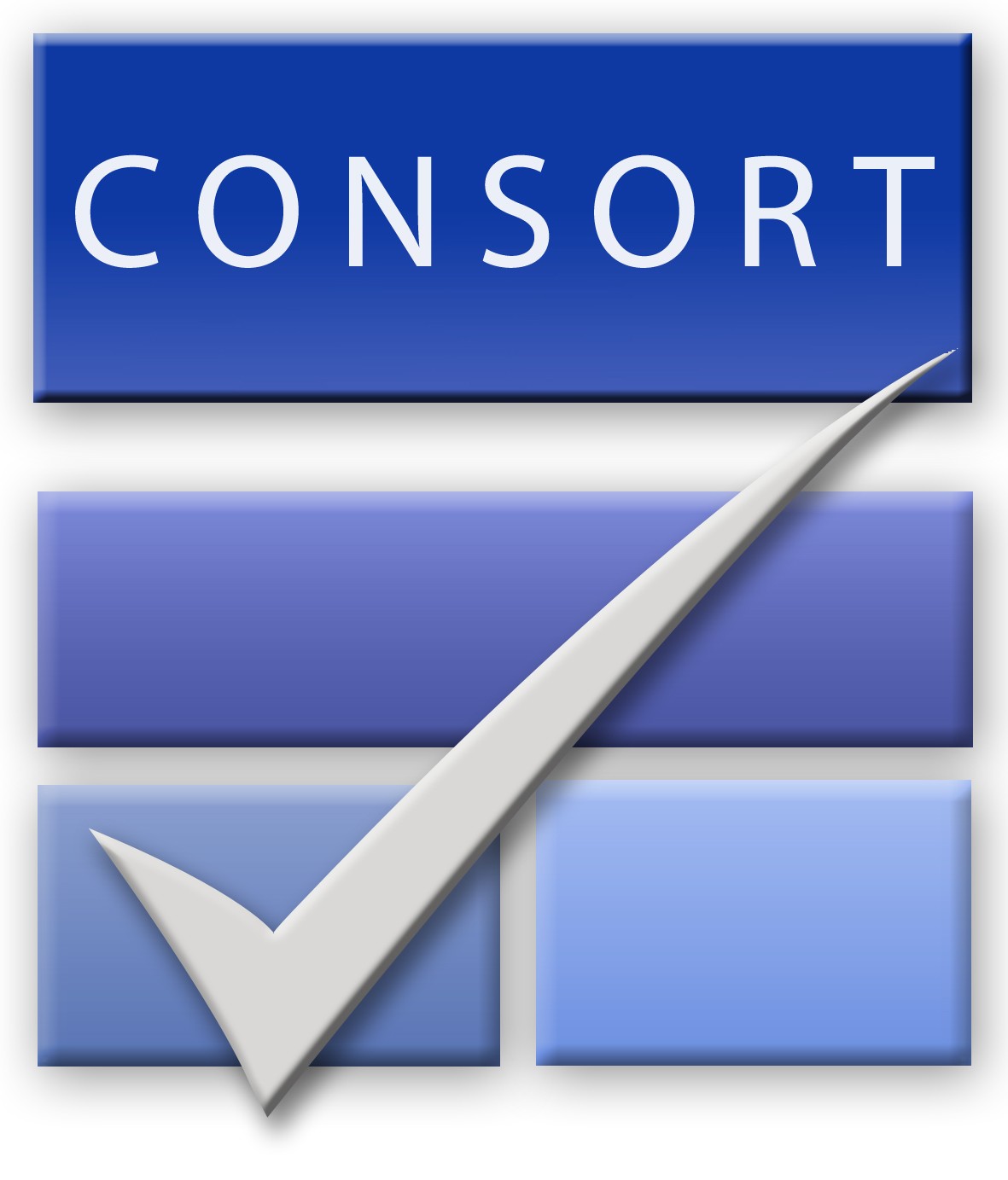
**CONSORT 2010 checklist of information to include when reporting a pilot or feasibility randomized trial in a journal or conference abstract**

| **Item** | **Description** | **Reported on line number** |
| --- | --- | --- |
| Title | Identification of study as randomised pilot or feasibility trial | Line 1 |
| Authors * | Contact details for the corresponding author | Lines 10-12 |
| Trial design | Description of pilot trial design (eg, parallel, cluster) | Lines 111-120 |
| Methods |  |  |
| Participants | Eligibility criteria for participants and the settings where the pilot trial was conducted | Lines 92-107 |
| Interventions | Interventions intended for each group | Lines 150-156 |
| Objective | Specific objectives of the pilot trial | Lines 85-88 |
| Outcome | Prespecified assessment or measurement to address the pilot trial objectives** | Lines 236-240 |
| Randomization | How participants were allocated to interventions | Lines 211-215 |
| Blinding (masking) | Whether or not participants, care givers, and those assessing the outcomes were blinded to group assignment | Lines 150-156 |
| Results |  |  |
| Numbers randomized | Number of participants screened and randomised to each group for the pilot trial objectives** | Lines 211-215 |
| Recruitment | Trial status† | Lines 92-93 |
| Numbers analysed | Number of participants analysed in each group for the pilot objectives** | Lines 217-233 |
| Outcome | Results for the pilot objectives, including any expressions of uncertainty** | Lines 244-311 |
| Harms | Important adverse events or side effects |  |
| Conclusions | General interpretation of the results of pilot trial and their implications for the future definitive trial | Lines 441-455 |
| Trial registration | Registration number for pilot trial and name of trial register | Lines 103-106 |
| Funding | Source of funding for pilot trial | Lines 441-455 |

Citation: Eldridge SM, Chan CL, Campbell MJ, Bond CM, Hopewell S, Thabane L, et al. CONSORT 2010 statement: extension to randomised pilot and feasibility trials. BMJ. 2016;355.

**this item is specific to conference abstracts*

***Space permitting, list all pilot trial objectives and give the results for each. Otherwise, report those that are a priori agreed as the most important to the decision to proceed with the future*

*definitive RCT.*

*†For conference abstracts.*
