# Supplementary material for: Evaluating the effects of embedded self-massage practice on strength performance: A randomized crossover pilot trial
Source: PLoS One. 2021 Mar 2;16(3):e0248031. doi: 10.1371/journal.pone.0248031 (PMC7924734; doi:10.1371/journal.pone.0248031)
Supplement: S4 File — CONSORT flowchart of the experimental procedures implemented for the pilot trial. (DOCX) [file pone.0248031.s004.docx]

Assessed for eligibility (n=14)

Excluded (n= 0)

♦  Not meeting inclusion criteria (n= 0)

♦  Declined to participate (n= 0)

♦  Other reasons (n= 0)

Assessed for objective 1 (n= 7)

Assessed for objective 2 (n= 7)

Lost to follow-up (give reasons) (n= 0)

Discontinued intervention (give reasons) (n= 0)

Allocated to SMR intervention (n= 7)

♦ Received allocated intervention (n= 7)

♦ Did not receive allocated intervention (give reasons) (n= 0)

Lost to follow-up (give reasons) (n= 0)

Discontinued intervention (give reasons) (n= 0)

Allocated to passive rest intervention (n= 7)

♦ Received allocated intervention (n= 7)

♦ Did not receive allocated intervention (give reasons) (n= 0)

Assessed for objective 1 (n= 7)

Assessed for objective 2 (n= 7)

## Allocation

## Assessment

## Follow-Up

Randomized (n= 14)

## Enrollment

Screened prior to eligibility assessment (n= 14)

Excluded (n= 0)

♦  Reasons (n= 0)

## Screened

## Follow-Up

Lost to follow-up (give reasons) (n= 0)

Discontinued intervention (give reasons) (n= 0)

Lost to follow-up (give reasons) (n= 0)

Discontinued intervention (give reasons) (n= 0)

Allocated to passive rest intervention (n= 7)

♦ Received allocated intervention (n= 7)

♦ Did not receive allocated intervention (give reasons) (n= 0)

Allocated to SMR intervention (n= 7)

♦ Received allocated intervention (n= 7)

♦ Did not receive allocated intervention (give reasons) (n= 0)

## Allocation
