# Supplementary material for: Evaluating the effects of embedded self-massage practice on strength performance: A randomized crossover pilot trial
Source: PLoS One. 2021 Mar 2;16(3):e0248031. doi: 10.1371/journal.pone.0248031 (PMC7924734; doi:10.1371/journal.pone.0248031)
Supplement: S5 File — Original information document provided to the participants (French version). (DOCX) [file pone.0248031.s005.docx]

**Document d’information**

Document d’information destiné aux personnes participant au projet de recherche impliquant la personne humain.

*Ce document a pour objectif de vous donner toutes les informations relatives à cette étude de façon à vous permettre d’exercer au mieux votre liberté de décision. Ce document est obligatoire et son contenu est*

*défini par le Code de la Santé Publique, article L 1122-1.*

*Il décrit précisément l’étude et mentionne toutes les autorisations réglementaires obtenues pour sa*

*mise en œuvre.*

*Vous devez conserver ce document. N’hésitez pas à poser des questions si vous ne comprenez pas*

*certains éléments.*

*La signature du formulaire de consentement n'affecte aucunement vos droits légaux.*

**Promoteur :** CAPSIX SAS, 19 ter rue Ampère, 69450 Saint-Cyr-Au-Mont-D’or, T. : 0670531162, [francois.eyssautier@capsix-robotics.com](mailto:francois.eyssautier@capsix.com)

**Investigateur principal : Pr. Aymeric GUILLOT**, Laboratoire Interuniversitaire de Biologie de la Motricité, Université Claude Bernard – Lyon 1, UFR STAPS – 27-29 Bd du 11 Novembre 1918**,** 069622 Villeurbanne Cedex, [aymeric.guillot@univ-lyon1.fr](mailto:aymeric.guillot@univ-lyon1.fr)

**Expérimentateur principal : Yann** KERAUTRET, Laboratoire Interuniversitaire de Biologie de la Motricité, Université Claude Bernard – Lyon 1. UFR STAPS – 27-29 Bd du 11 Novembre 1918**,** 069622 Villeurbanne Cedex[, yann.kerautret@capsix-robotics.com](mailto:yann.kerautret@capsix-robotics.com)

**Lieu de la recherche :** Plateforme d’expérimentation IRMIS (Institut Régional de Médecine et d’Ingénierie du Sport) – Campus Santé Innovation de l’Université Jean Monnet, 10 Rue de la Marandière, 42270 Saint-Priest-en-Jarez.

**Titre de la recherche :** Évaluation de l’efficacité d’une routine d’automassages inter-séries dans le développement de la force.

Madame, Monsieur, nous vous proposons de participer à une recherche clinique sur volontaires sains (30 participants seront recrutés au total). Le protocole de recherche auquel nous vous proposons de participer est le suivant : il se déroulera lors de 2 sessions programmées entre 14h et 18h, au sein de la Plateforme technologique de l'Institut Régional de Médecine et d'Ingénierie de Sport (IRMIS), située sur le Campus Santé Innovation de l’Université Jean Monnet.

Le protocole expérimental sera, pour chaque session, constitué d’un pré-test, d’une condition expérimentale et d’un post-test. Vous participerez aux conditions expérimentales suivantes : automassage, condition contrôle. Chaque session durera 1h.

- Lors de la condition ‘automassage’, nous vous demanderons de réaliser un automassage à l’aide d’un rouleau de massage et d’un bâton de massage, avec ou sans roulement. La routine d’automassage sera standardisée en ciblant le quadriceps. Toutes les séances s’effectueront systématiquement sous la supervision du même expérimentateur, spécialiste en la matière, pour garantir la sécurité et la bonne exécution du protocole.
- Lors de la condition ‘contrôle’, vous serez invité(e) à rester au repos sans engager d’activité spécifique.

# Recrutement des participants volontaires

- *Critères d'inclusion* :
- Pour participer à cette étude, vous devez être âgé(e) de 18 ans minimum et de 50 ans maximum, être affilié(e) à un régime de sécurité sociale, donner votre consentement libre par écrit.
- Vous ne pouvez être inclus(e) dans plusieurs protocoles de recherches biomédicales simultanément. Si vous participez à une autre étude, vous ne pourrez participer à ce protocole avant l’expiration de la période d’exclusion de l’étude précédente.
  - *Critères de non-inclusion*
- Grossesse
- Affections cutanées en phase aigüe sur les membres inférieures et le dos
- Troubles cardiovasculaires et pathologies respiratoires, orthopnée, insuffisance cardiaque, hypertension labile ou non équilibrée par un traitement, troubles du rythme cardiaque, port d’un pacemaker
- Insuffisance artérielle ou veineuse connue (Athérosclérose ou suspicion de thrombose veineuse), ulcères variqueux et varices douloureuses
- Antécédents neurologiques (comitialité, altération du jugement ou nécessitant la prise de substances neuroactives (hypnotiques, antiépileptiques, psychotropes, myorelaxants)
- Antécédent traumatique sévère des membres inférieurs et maladies neuromusculaires chroniques
- Trouble musculo-tendineux affectant le dos et les membres inférieurs de moins de 3 mois

***Méthodologie***

Chaque condition expérimentale débutera par la présentation du déroulement de l’expérience à l’aide d’une fiche d’instructions.

L’investigateur ou l'expérimentateur qui vous accueillera vous expliquera les différentes étapes. Chaque pré-test comprend :

- Des mesures à l’aide de questionnaires
- Des mesures de performance en force

Les sessions seront programmées en journée entre 14-18h.

# Risques prévisibles

Il n’y a aucun risque spécifique lié au protocole de recherche, qui utilise des mesures externe, non invasive.

# Modalités d’information

Les instructions pour la réalisation des tâches expérimentales vous seront fournies et expliquées en détail par l’expérimentateur. À tout moment, en dehors des périodes d’enregistrement, vous pourrez lui demander des précisions.

# Indemnité

Vous percevrez une indemnité de 20€ pour votre participation à l’étude complète.

# Procédures relatives à la protection des personnes

En application de l'article L 1121-4 du Code de la Santé Publique, cette recherche biomédicale a obtenu un avis favorable du CPP 2019-A01732-55 et l'autorisation de l’autorité compétente CPP 1223 HPS2.

Conformément au Code de la Santé Publique, CAPSIX SAS en tant que promoteur a souscrit un contrat d'assurance auprès de la compagnie AXA (Contrat n°10447510404 – Siège social : 313 Terrasses de l’Arche, 92727 NANTERRE Cedex).

Votre participation sera recensée sur le "Fichier national des personnes qui se prêtent à des recherches biomédicales" (article L 1121-16 du code de la santé publique).

Cette étude ne comporte pas de période d’exclusion. Vous pourrez donc participer à une autre étude dès le lendemain de votre examen EEG.

Les données recueillies dans le cadre de cette étude feront l’objet d’un traitement informatisé et anonymisé selon les procédures et traitement des données conforme à la méthodologie de référence MR001 éditée par la CNIL et conformément à la loi 78-17 du 06 janvier 1978 relative à l’informatique, aux Fichiers et aux Libertés, modifiée par la loi n° 94-548 du 1er juillet 1994, relative au traitement des données nominatives ayant pour fin la recherche dans le domaine de la santé. Les données seront archivées au sein du LIBM pendant 25 ans.

Conformément aux dispositions de loi relative à l’informatique aux fichiers et aux libertés (CNIL), vous disposez d’un droit d’accès et de rectification et d’un droit d’opposition à la transmission des données couvertes par le secret professionnel susceptibles d’être utilisées dans le cadre de cette recherche et d’être traitées. Vous disposez également d’un droit d’obtenir, de la part du responsable de l’étude, de l’effacement de données à caractère personnel. Si vous jugez que l’étude a porté atteinte aux règles de protection de vos données personnelles, vous pouvez adresser une réclamation à la CNIL.

Vous pouvez également accéder directement ou par l’intermédiaire d’un médecin de votre choix à l’ensemble de vos données médicales en application des dispositions de l’article L 1111-7 du Code de la Santé Publique.

Ces droits s’exercent auprès du médecin qui vous suit dans le cadre de la recherche.

La publication des résultats de la recherche ne comportera aucun résultat individuel identifiant ou non anonyme. Elles sont la propriété des investigateurs (Pr. Aymeric Guillot), et sont traitées confidentiellement. Elles pourront faire l’objet de présentations scientifiques ou de publications dans la presse médicale et scientifique, mais dans aucun cas votre nom ne figurera avec les données. Pendant l’étude, un numéro ainsi qu’un code constitué des trois premières lettres de votre nom et des deux premières lettres de votre prénom vous seront attribués. Leur consultation n'est autorisée que par les responsables de l'étude et par un représentant des autorités de santé.

Vous avez la possibilité d'être informé des résultats globaux en fin l'étude, conformément au dernier alinéa de l'article L1122-1.

Si vous décidez d’interrompre l’étude, sachant que votre participation à celle-ci est totalement libre et que vous pouvez quitter l’essai à tout moment, sans subir aucun préjudice, il vous sera demandé d’en informer rapidement le médecin investigateur. Vous recevrez alors une indemnité au prorata de votre participation.
