# Supplementary material for: Evaluating the effects of embedded self-massage practice on strength performance: A randomized crossover pilot trial
Source: PLoS One. 2021 Mar 2;16(3):e0248031. doi: 10.1371/journal.pone.0248031 (PMC7924734; doi:10.1371/journal.pone.0248031)
Supplement: S6 File — Original information document provided to the participants (English translation). (DOCX) [file pone.0248031.s006.docx]

**Information document**

The present information document aims at informing the participants to the present research project.

The purpose of this document is to provide you with all the information relating to this study so as to allow you to exercise your freedom of decision as best as possible. This document is mandatory and its content is defined by the Public Health Code, article L 1122-1.

It precisely describes the study and contains all legal mentions necessary to conduct this research. You must keep this document. Don't hesitate to ask questions if you don't understand certain elements.

Signing the present written informed consent form does not affect your legal rights. Promoter: CAPSIX SAS, 19 ter rue Ampère, 69450 Saint-Cyr-Au-Mont-D’or, T.: 0670531162, francois.eyssautier@capsix-robotics.com

Principal investigator: Pr. Aymeric GUILLOT, Interuniversity Laboratory of Motricity Biology, Claude Bernard University - Lyon 1, UFR STAPS - 27-29 Bd du 11 Novembre 1918, 069622 Villeurbanne Cedex, aymeric.guillot@univ-lyon1.fr

Main experimenter: Yann KERAUTRET, Interuniversity Laboratory of Motricity Biology, Université Claude Bernard - Lyon 1. UFR STAPS - 27-29 Bd du 11 Novembre 1918, 069622 Villeurbanne Cedex, yann.kerautret@capsix-robotics.com

Place of research: IRMIS experimentation platform (Regional Institute of Sports Medicine and Engineering) - Jean Monnet University Health Innovation Campus, 10 Rue de la Marandière, 42270 Saint-Priest-en-Jarez.

Research Title: Evaluating the Effectiveness of an Inter-Set Self-Massage Routine in Strength Development.

You volunteered to participate to a clinical research protocol on healthy volunteers (30 participants will be recruited in total). The research protocol will unfold as follows: you will participate to 2 sessions, scheduled between 2 p.m. and 6 p.m., within the technological platform of the Regional Institute of Medicine and Sports Engineering (IRMIS), located on the Health Innovation Campus of Jean Monnet University.

The experimental protocol will, for each session, consist of a pre-test, an experimental condition and a post-test. You will participate in the following experimental conditions: self-massage, control condition. Each session will last 1 hour.

- During the "self-massage" condition, we will ask you to perform a self-massage using a massage roller and a massage stick, with or without rolling. The self-massage routine will be standardized and target quadriceps muscles. All sessions will be carried under the supervision of the same experimenter, specialist in the field, to guarantee the safety and the correct execution of the protocol.

- During the "control" condition, you will be asked to remain at rest without engaging in any specific activity.

Recruitment of volunteer participants

• Inclusion criteria:

- To participate in this study, you must be at least 18 years old and at most 50 years old, be affiliated with a social security scheme, give your free written consent.

- You cannot be included in several biomedical research protocols simultaneously. If you are participating in another study, you will not be able to participate in this protocol until the exclusion period of the previous study has expired.

• Non-inclusion criteria

- Pregnancy

- Acute skin conditions on the lower limbs and back

- Cardiovascular disorders and respiratory pathologies, orthopnea, heart failure, labile hypertension or hypertension not balanced by treatment, heart rhythm disturbances, wearing a pacemaker

- Known arterial or venous insufficiency (Atherosclerosis or suspicion of venous thrombosis), varicose ulcers and painful varicose veins

- Neurological history (comitiality, impaired judgment or requiring the intake of neuroactive substances (hypnotics, antiepileptics, psychotropic drugs, muscle relaxants)

- Severe traumatic history of the lower limbs and chronic neuromuscular diseases

- Musculotendinous disorder affecting the back and lower limbs less than 3 months old

Methodology

Each experimental condition will begin with the presentation of the conduct of the experiment using an instruction sheet.

The investigator or experimenter who will welcome you will explain the different steps to you. Each pre-test includes:

- Measurements using questionnaires

- Strong performance measurements

The sessions will be scheduled during the day between 2 pm-6pm.

Foreseeable risks

There is no specific risk associated with the research protocol, which uses external, non-invasive measures.

Information methods

Instructions for performing the experimental tasks will be provided and explained in detail by the experimenter. At any time, outside of check-in periods, you can ask for details.

Indemnity

You will receive compensation of € 20 for your participation in the full study.

Procedures relating to the protection of persons

Pursuant to article L 1121-4 of the Public Health Code, this biomedical research obtained a favorable opinion from the CPP 2019-A01732-55, and authorization from the competent authority CPP Ouest 6 – CPP 1223 HPS2.

In accordance with the Public Health Code, CAPSIX SAS as promoter has taken out an insurance contract with the company AXA (Contract n ° 10447510404 - Headquarters: 313 Terrasses de l'Arche, 92727 NANTERRE Cedex).

Your participation will be listed on the "National file of people who lend themselves to biomedical research" (article L 1121-16 of the public health code).

This study does not include an exclusion period. You can therefore participate in another study the day after your EEG examination.

The data collected as part of this study will be subject to computerized and anonymized processing according to the procedures and data processing in accordance with the MR001 reference methodology published by the CNIL and in accordance with Law 78-17 of January 6, 1978. relating to computers, files and freedoms, amended by law n ° 94-548 of 1 July 1994, relating to the processing of personal data for the purpose of research in the field of health. The data will be archived within LIBM for 25 years.

In accordance with the provisions of the law relating to data processing, files and freedoms (CNIL), you have a right of access and rectification and a right of opposition to the transmission of data covered by professional secrecy likely to be used in the context of this research and to be processed. You also have a right to obtain, from the study manager, the erasure of personal data. If you believe that the study has violated the rules for the protection of your personal data, you can file a complaint with the CNIL.

You can also access all of your medical data directly or through a doctor of your choice in accordance with the provisions of Article L 1111-7 of the Public Health Code.

These rights are exercised with the doctor who follows you as part of the research.

The publication of research results will not include any identifying or non-anonymous individual results. They are the property of the investigators (Pr. Aymeric Guillot), and are treated confidentially. They may be the subject of scientific presentations or publications in the medical and scientific press, but under no circumstances will your name appear with the data. During the study, you will be assigned a number and a code consisting of the first three letters of your name and the first two letters of your first name. Their consultation is only authorized by those in charge of the study and by a representative of the health authorities.

You have the possibility of being informed of the overall results at the end of the study, in accordance with the last paragraph of article L1122-1.

If you decide to interrupt the study, knowing that your participation in it is completely free and that you can leave the trial at any time, without suffering any damage, you will be asked to promptly inform the investigating doctor. . You will then receive compensation in proportion to your participation.
