# Supplementary material for: Evaluating the effects of embedded self-massage practice on strength performance: A randomized crossover pilot trial
Source: PLoS One. 2021 Mar 2;16(3):e0248031. doi: 10.1371/journal.pone.0248031 (PMC7924734; doi:10.1371/journal.pone.0248031)
Supplement: Attachment — Submitted filename: response_to_reviewers.docx [file pone.0248031.s007.docx]

Dear Editor,

Thank you for your consideration and for the time and efforts invested into the evaluation of our manuscript. The constructive and in-depth comments of the reviewers were much appreciated, and we appreciate the opportunity to proceed with a revised version addressing their comments and queries. We agreed on most issues underlined in their reviews, and revised our manuscript accordingly. All remarks were addressed in the point-by-point answer file, and yielded to substantial modifications appearing in red in the revised manuscript with tracked changes. We paid specific attention to the structure and gist of the introduction and methods sections, and included several clarifications regarding the statistical procedures. We remain open to any further modification susceptible to improve the quality of this work.

On behalf of all co-authors,

Franck Di Rienzo

**Comments to the Author**

Reviewer #1: The manuscript presents an analyses of a randomized cross-over trial, aimed at examining the intervention effects of a foam rolling intervention. The study was IRB approved, however, I couldn't find the appropriate registration in clinicaltrials.gov (with a specific number) or within some other entity. If I am correct, it's not mentioned in the manuscript. The design looks adequate, however, I still have some questions on the design aspects of the study.

We gratefully acknowledge Reviewer 1 for the time and effort put into the evaluation of the original version of our manuscript, and the constructive inputs to enhance the quality of our work. Reviewer 1 is right, the study was ethically approved by our local institutional review board but was not registered on an online clinical trial platform. This is due to the atypical nature of sports science research designs, which involves human participants and longitudinal designs without being associated with risks/adverse health effects compared to drug administration. As mentioned by the Editor in the decision letter, this represents a grey area. While this can be debated, examining sports science experiments reveals very limited registration on online platforms. Sports science research abide somehow distinct standards from clinical trials in medicine. We hope that reframing the present work as a pilot study will help to overcome this issue.

We amended the title of the revised manuscript to attenuate potential confusions with randomized controlled trials in medicine (PAGE 1). The CONSORT checklist and flowcharts were also amended, and we included as supplementary files their versions for pilot trials. If the Editor and/or Reviewer 1 deem it necessary, we could prospectively register the present study to an online platform for absolute transparency.

1. Sample size statement: With only n = 14 participants, I would be interested to know in advance what power one can expect in the analyses with some pragmatic effect size. The writeup states: "Fourteen CrossFit athletes volunteered to participate...". You cannot conduct and analyze a clinical trial, unless it is sufficiently powered. Recruitment is expected to continue until the desired sample size is reached within a pragmatic time-frame. Else, it can be called a simple "pilot trial" designed to evaluate some initial effectiveness of an intervention. Again, it's not clear from the writeup. The authors should present a section on power/sample size considerations. There maybe R packages available for conducting power analyses under cross-over designs.

Reviewer 1 raises here an important concern regarding the methodological design. We agree that power considerations are crucial when designing randomized controlled trials, particularly to determine the appropriate sample size. While we did not perform a priori power analyses to determine the ideal number of participants needed for the experiment, we initially determined the sample size based on previous studies addressing the efficacy of self-myofascial release interventions, some of which involved cross-over experimental designs (Hodgson, 2019, doi: 10.1519/JSC.0000000000002271; Peacock, 2015; doi: 10.1519/JSC.0000000000000867: Behm, 2018; doi: 10.25036/jphr.2018.2.1.behm). Implementing a crossover design was expected to increase the statistical power. Indeed, between-subjects designs are more affected by inter-individual variability. Large sample sizes are thus required to generate reliable data and reveal genuine patterns, while within-subjects designs offer increased statistical power to detect robust (i.e. systematic) variations in the dependent variables. Anecdotally, a recent study suggests that between-subjects designs require 4 to 8 times more participants than within-subjects designs (Bellemare et al., 2014). Another psychology study comparing effect sizes of within- and between-subjects designs demonstrated that even with half the number of participants, a within-subjects design was much more powerful than its between-subjects counterpart (Thompson and Campbell, 2004). These a priori considerations were expected to overcome potential statistical power limitations. Nonetheless, we agree with Reviewer 1 that referring to a “randomized controlled trial” was incorrect, and potentially misleading. We agree with his/her suggestion that “pilot trial” is more appropriate, as we indeed intended to provide an initial effectiveness evaluation of an embedded self-myofascial release intervention.

In the revised manuscript, we deleted the term “randomized crossover trial” from the title to avoid any approximation regarding the methodology. As suggested, we now introduce the work as a “pilot trial”. In keeping with his/her recommendation regarding power and sample size calculations, we included a power/sample size section in the methods of the revised manuscript (PAGE 12). We used the pwr package (Champely, 2018; Package ‘pwr.’ Retrieved from <http://cran.r-project.org/package=pwr>) to run a posteriori power calculation based on the effect sized yielded by the linear mixed effects analysis and the type 1 statistical significance threshold. We amended the methods and results sections of the revised manuscript accordingly (PAGES 12-15). The revised paper now clearly provides pilot results under the form of effect sizes obtained for n = 14 (sample size), their statistical significance and the corresponding power (p_1-β ;_ see Result section).

2. Writeup. The study has been submitted as a Research Article, and not a clinical trial. I am wondering, if the authors can refer clearly to the main clinical trial paper (already?) published from the randomized trial. Now, if that has not happened, the current writeup needs a thorough change, reflecting the CONSORT guidelines for submission of a clinical trial and it's analyses, and submitted as a Clinical Trial.

Reviewer 1 is correct. This work cannot be considered a randomized control trial, and there is no main clinical trial paper. Reviewer 1’s comments are a clear indication of expertise in medical research and designing randomized controlled trials for clinical interventions. We agree that the writeup must be amended throughout the revised manuscript to better reflect the nature of our work.

In the revised manuscript, we removed the terms indicative of the clinical nature of the intervention. As suggested by Reviewer 1 in his/her preceding comment, the work should better be considered “pilot trial”. We amended the writeup accordingly, ensuring that it remained consistent throughout the revised manuscript (e.g. methods and discussion sections).

3. Randomization. Block randomization was used. What was the block size?

Block randomization was used to assign a condition order for the Roller Massager and Control experimental conditions. After poster advertising and direct contact between coaches and practitioners at the Crossfit facility, 8 males and 6 females manifested their interest to participate in the experiment (PAGES 5; 11). To complete the block randomization and assign an order for experimental conditions to the participants (Roller massager first, Control first), we used the R *blockrand* package. *Blockrand* allows block randomization with random block size selection to prevent randomization bias (Efrid, 2010; doi: [10.3390/ijerph8010015](https://dx.doi.org/10.3390%2Fijerph8010015)). Here, with 2 levels of random assignment, the actual block sizes were randomly chosen from the set (2, 4, 6, 8) (<http://127.0.0.1:11525/library/blockrand/html/blockrand.html>). To obtain the exact number of randomized males and females, i.e. corresponding to the number of our volunteering participants’, we used a “while” loop in R controlling for the length of the *blockrand* dataset output:

Figure 1. R script used for block randomization

This yielded two blocks for males (n = 6, n= 2), and one block (n = 6) in females (replicated below for the purposes of illustration; Fig. 2).

Fig. 2. Block randomization (illustration of *blockrand* output)

In the revised manuscript, we included more detailed information regarding block sizes and the above-mentioned reference for greater transparency and reproducibility (PAGE 11).

4. Statistical Analysis: Linear mixed effects, via R nlme, was used. How can one guarantee the Gaussian (Normal) behavior of the response variable? Was it checked?

We omitted to indicate the statistical procedures implemented to assess models validity, and thank Reviewer 1 for underlining the need for clarifications. Unless we are incorrect, it seems that the normal distribution of the residuals has more relevance with regards to the validity of the linear mixed effects model than the Gaussian behavior of the response variable. We thus followed the guidelines indicated in Winter (2013, Linear models and linear mixed effects models in R with linguistic applications. arXiv:1308.5499. [http://arxiv.org/pdf/1308.5499.pdf]) to assess validity of the linear and linear mixed effects models. We performed visual inspections of the residual plots and found no departures from the assumptions of normality or homoscedasticity (ex. Fig 2, for an illustration).

Fig. 3. Residual plots for the linear mixed effects analysis carried on concentric power data

This information was included in the revised manuscript (PAGE 11). If necessary, we could perform a clustered analysis of the Gaussian behavior of all response variables and amend the revised manuscript accordingly.

5. Conclusions/Discussions: With n = 14, the study results can at best be reported as a very pilot study! This needs to be clearly mentioned.

We agree with Reviewer 1. Our original phrasings did not emphasize enough limitations related to sample size limitations inherent to the pilot nature of this study.

In the revised manuscript, we included "pilot study" in the title. We explicitly mention that the experiment provides primarily pilot results that need further investigation in more robust experimental designs (PAGES 15-16; 21). In keeping with Reviewer 1’s comment, we deemed important to explicitly incorporate statements of “very pilot results” in the discussion section (PAGE 19).

Reviewer #2: I happen to have reviewed this manuscript before and I have to say this is a much improved version. I have a few minor comments regarding interpretation and language use to hopefully improve accuracy and readability of the manuscript – these can be found below:

We thank Reviewer 2 for his/her acceptance to endorse the expertise of our manuscript for the second time. We were thus particularly sensitive to the encouraging feedback regarding the efforts put into improving the quality of our work. We also thank Reviewer 2 for the careful reading and underlining of remaining typos, and for systematically highlighting issues that required clarifications and/or corrections.

All Reviewer 2 comments and queries yielded substantial changes in the revised manuscript, and are underlined in the point-by-point answer to below.

Introduction:

It is, to some extent, unclear what the first two paragraphs of introduction are trying to achieve. They would read completely fine if this was a literature review. However, here, you are trying to introduce your experiment. It is thus unclear how the previous work presented in the first two paragraphs links to the present work – this becomes somewhat clearer later on in the introduction; but for readability sake, it would be much better if the reader didn’t have to make such connection themselves (in fact, a reader that is more naïve to the literature might struggle to make the connection).

We agree that the first two paragraphs were too vague, creating a discontinuity between the first and the second part of the introduction. As suggested, this first part deserves to be refocused on our experiment. Refocusing the introduction on key components leading to the experimental hypotheses should contribute to improve its conciseness and focus, and avoid misleading the readership.

The first part of the introduction was amended accordingly (PAGES 3-4). All statements in the introduction section of the revisited manuscript were carefully reformulated following the major commentary in Reviewer 2.

Line 45: consisting ‘of’.

Line 52: ‘has been’ associated.

Line 54: static stretching should be singular (currently written as ‘statics’).

Line 56: ‘might participate’ is a strange term, please revise. I’m not entirely sure what you are trying to say in this sentence, otherwise I would suggest the alternative. Also, ‘athletic performance’ should be singular.

Line 57: Do you mean ‘repeated sprint performance’?

Line 63: ‘have been hypothesised’.

Line 63-66: How is this relevant to your investigation? Again, see my major comments about the Introduction, but this is a clear example of that.

Line 74: Do you mean ‘Monteiro et al.’?

All typos were corrected in the revised manuscript:

- We corrected grammatical errors underlined by Reviewer 2.
- As suggested, we used the terms "athletic performance" and "repeated sprint performance" instead of "physical performances" and "agility sprints repetition".
- Following Reviewer 2’s remark and going through a thorough review of our manuscript, we decided to delete lines 63-66 to gain clarity.
- We have corrected authors’ names in the quote line 74.

Methods:

Line 138: I believe you have forgotten to translate ‘March’ from French into English.

Reviewer 2 is right. This was amended in the revised manuscript (PAGE 8).

Results:

Line 224: Did this differ statistically between groups?

Line 229: I believe ‘were greater’ is missing.

Line 254: Have you calculated measurement error for thigh circumference? Given the difference is quite small, it is important to establish a measurement error so the meaningful effect can be established (see some work by Monteiro, 2017; <http://www.ncbi.nlm.nih.gov/pmc/articles/PMC5294949/> that showed the lack of meaningful difference even when the difference was statistically significant).

Line 268: Please reconsider the expression – ‘there WAS a significant interaction’ is much more appropriate (there is no such thing as an effect of an interaction when reporting results of the null-hypothesis significance testing).

Line 272: How can you be sure it approached significance and was not moving away from it? Please rephrase.

We thank Reviewer 2 for the careful reading and for systematically highlighting issues that required clarifications and/or corrections:

- Differences were statistically significant for the number of set, but not for the number of reps per set (PAGE 12). Reviewer 2’s comment also enabled us to update minor mistakes in the reports of mean, sd and R^2^_p_values (NB. the raw data file submitted as supplementary file remains unchanged). We amended the methods and results sections accordingly in the revised manuscript.
- We rectified the omission L. 229.
- We did not consider measurement error, which could indeed have been relevant to establish the practical significance of statistically significant differences. This represents a clear limitation in the original manuscript, although the results pattern for thigh circumference appeared congruent with those observed for the other dependent variables (PAGES 13-14; 17). An overview of the literature data related to waist circumference measurement revealed that 5% could be considered a rule of thumb for the measurement error for waist circumference (Verwieij, 2013; doi: [10.1017/S1368980012002741](https://www.researchgate.net/deref/http%3A%2F%2Fdx.doi.org%2F10.1017%2FS1368980012002741?_sg%5B0%5D=mteWMIqLPoqRV-VGn4mCnKp1kbur27UIQQMQBebGbsYFc1vyaHBxApJSFUVHupkvQoMZbdxCYX3yZN-LURF19-YL7A.UN0_GFQ_OkhqXmLvBIMRVSFtrIm2uNNc1uWkSbVNyOCYF2YtsKEs37wLPtzTQDbWNN22ZeekUGi-Gj8pQhndYQ)). The pretest versus posttest differences remained below the 5% range. At the meantime, departures higher than 5% from the pretests after a single session would have been surprising. As a counterpoint, thigh circumference measurements were conducted by the same experimenter. This indicates that the distribution of the error across tests and experimental conditions was homogenous. Unilateral and systematic deviations within the measurement error range would have been required to bias the results pattern. This information was included in the revised manuscript, and we included additional references, including those emphasized by Reviewer 2, for purposes of transparency.
- We agree that the phrasing was incorrect. It could very well be indicative of a departure trend in case of higher sampling. We neutrally state that the effect “failed” to reach the statistical significance threshold in the revised manuscript (PAGE 14).

Discussion:

Line 302: consisted ‘of’.

Lines 305-306: You could provide the percentage change values in the brackets in the previous sentence; currently you are merely repeating the same information (with the exception of the quantification of a decrease) from the previous sentence, making text redundant and repetitive.

Line 320-321: I suggest this sentence be rephrased. The expression to ‘compound the physical fatigue elicited by the resistance training protocol’ is unclear. Do you mean ‘could facilitate fatigability elicited by the resistance training protocol?’. Note that I also used the term ‘fatigability’ rather than ‘fatigue’ – the former describes an objective decline in a measure of muscle function during a discrete task, whereas the latter is a perception or feeling of exhaustion (see Enoka & Duchateau 2016 Med Sci, for clear distinction in terminology).

Line 321-323: There is no evidence for such a claim. In fact, research suggests that pressure far outside the physiological threshold are required to induce mechanical changes to tissue with foam rolling (see Chaudhry et al. 2008 J Am Osteopath Assoc).

Line 339-340: The baseline VAS scores being zero more likely indicates the lack of muscle damage/complete recovery than the lack of ‘residual muscle fatigue’. “Muscle fatigue” (or more appropriately termed ‘fatigability’) is a transient reduction in muscle force output that recovers withing minutes/hours of exercise. If the reduction in muscle force is persistent for days, it is typically classed as muscle damage (see Carroll et al. 2017, J Appl Physiol).

Line 371: ‘Physical’ seems a strange adjective for fatigue. Fatigue in its true sense of a word is a psychophysical phenomenon and one cannot necessarily be distinguished from the other (again, see Duchateau & Enoka 2016).

Line 371: It ‘has previously been shown’.

Line 377-378: The role of muscle damage in muscle hypertrophy is a debatable mechanism; e.g. mechanical signalling and metabolic stress are much more prominent triggers of the muscle remodelling process (see Wackerhage et al. 2019, J Appl Physiol).

Again, we thank Reviewer 2 for emphasizing the points that required corrections/clarifications throughout the discussion section of the revised manuscript. We paid specific attention to the recommended citations, which we agree will help clarifying the scientific background of discussion statements. The distinction between the terms "fatigability" and "fatigue" is judiciously suggested, and was made throughout the revised manuscript. Please find below a point-by-point answer.

- We fixed the typos (e.g. errors line 302 and line 371)
- We provided the percentage change into brackets lines 322-324, as recommended.
- We rephrased our sentence Line 320-321. We agree that the terminology suggested by Reviewer 2 adds clarity to the discussion. We incorporated the suggested reference for readers interested in the fundamental distinctions between the concepts of “fatigue” and “fatigability” (PAGES 17; 19).
- We agree that our original statements could be misleading and required rephrasing. We intended to convey the idea that high pressure levels during foam rolling (which are generally superior to those applied during manual therapy; Freiwald, 2016; doi: 10.1016/j.orthtr.2016.07.001, doi: 10.1016/j.orthtr.2016.07.002, PAGE 17), may be a cause for adverse effects on performance (e.g. micro-damage to the muscles altering their short-term contractile properties). We thus rephrased our initial statements, and contextualized our postulates within the framework underlined by Reviewer 2 – i.e. indicating that pressures are actually crucial to elicit hormesis-type response patterns. We also included the citation suggested by Reviewer 2, although it seems that it did not involve research on foam rolling but on the effect of manual massage therapy (PAGE 17).
- We agree that referring to an "absence residual muscle fatigue" was inaccurate. Referring to “the lack of muscle damage” is more accurate, since it distinguishes more clearly fatigue (transient, as indicated by Reviewer 2) from muscle recovery (persistent, also indicated by Reviewer 2) processes. We included the suggested reference to clarify the scientific background supporting the claims made when interpreting VAS scores for muscle damage in the revised manuscript (PAGE 18).
- We agree that "psychophysical" is more accurate, and better fits the conceptual frameworks developed throughout the discussion (i.e. psychobiological models; PAGE 19). This was amended in the revised manuscript.
- We share the nuanced opinion of Reviewer 2 regarding the mechanisms of muscle hypertrophy, and appreciate the recent reference provided in his/her comment. Therefore, we decided to delete lines 377-378. If Reviewer 2 deems it preferable, we could alternatively nuance our statement and include the reference suggested as scientific rationale to temper our claims.

The conclusion of the study seems rather abrupt. It would be prudent to briefly summarize the main findings (in a sentence or two) and infer meaning from it (e.g. functional consequence).

We agree and amended the conclusion according to the suggested framework. We now provide a brief summary of the pattern of findings, and outline main functional/consequence/practical implications/research perspectives (PAGE 21).

Reviewer #3: Below are some comments:

We thank Reviewer 3 for accepting to review our manuscript and his/her constructive evaluation. All comments were taken into account in the revised version. Please find below the point-by-point answer to each comment and query.

In Introduction more attention should be paid on explaining why foam rolling could influence on muscle performance in strength training as you hypothesized that embedded foam rolling with a roller massager would reduce the strength performance.

We agree that further background regarding the potential benefits of embedded foam rolling practice on strength should be provided in the introduction. In the revised manuscript, we thus paid specific attention to explain why foam rolling could influence muscle performance in strength training (PAGE 4).

Line 46: Author’s wrote: (…) Foam rolling can be administered using a foam roller, a roller massager, sticks or balls of varying sizes and densities (1,3,4)”. If you are writing foam rolling it determines specific tool known as foam roller. Of course, you can use different equipment like you’ve mentioned but it wouldn’t be foam rolling. I think you meant Self Myofascial Release technique.

We thank Reviewer 3 for underlining these necessary distinctions regarding to the terminology. It is true that foam rolling belongs to the category of “Self-Myofascial Release Techniques”. It is also true that the tool used in this study is a roller massager, and not a foam roller. Hence, the term "foam rolling" was inappropriate.

In the revised version, references to “foam rolling” were systematically deleted, and the revised manuscript refers to Self-myofascial release using a roller massager throughout the introduction, methods and discussion sections of the revised manuscript.

Line 51 and later in the text: please provide next numbers order

We thank Reviewer 3 for the careful reading, we amended this error and performed verifications that no such errors remained throughout the revised manuscript.

I don’t know why you compare male and females in the same group, as the training effects, somatic build, recovery capacity and many other factor significantly differ both sexes?

Reviewer 3 raised here an important issue. We admit that we did not differentiate between men and women regarding inclusion criteria. Since inclusion criteria were restrictive, particularly the need for experience in weightlifting and self-myofascial release, all participant (men and women confounded) who expressed interest to participate in the experiment were enrolled in the design. This yielded a limited sample (n = 14), which we did not further subcategorize by gender. Rather, we used a repeated measures design controlling for inter-individual variability. A possible way to address Reviewer 3’s query would be controlling for the effect of GENDER when designing the linear mixed effects analysis. This, however, goes beyond the original focus of this pilot study, and would require a larger sample of participants.

From an applied perspective, maintaining a mixed gender sample could also have practical relevance. This indicates that these pilot results were obtained irrespective of the effect of gender. At the meantime, Reviewer 3 is right that gender differences should be expected in larger sample of participants, although these may not interact with the effect of massage intervention (e.g. Skinner B, doi:10.1016/j.jbmt.2020.01.007; Cavanaugh MT

doi:10.1519/JSC.0000000000001625). We included these references to discuss the limitation of the pilot trial with regards to the control of gender differences. We explicitly included this limitation in the discussion section of the revised manuscript, and underline that this issue goes beyond the initial focus of the pilot study and would ideally require a higher sample of participants (PAGE 16). If Reviewer 3 remains unconvinced, we could perform a new data analysis accounting for the effect of gender and rewrite the results section (since all values would differ with the inclusion of an additional factor). A preliminary investigation reveals that while the expected main effect is present, it does not interacts with the effect of experimental condition, hence leaving the results pattern unchanged (see Figure 4).

Fig 4. .Summary of the Anova table for the linear mixed effects analysis including the effect of GENDER carried on total workload data.

Could the authors add the sample size? The description of the studied group lacks information on the sampling methodology. The authors do not state whether the selection was random or different, there is also no information about the sampling frame, as well as the method of reaching particular groups or participants - please add this information. It also needs to be clarified in the paragraph regarding study population how the participants were identified. Are the participants representative for a larger group of CrossFit Athletes or is it a very specific group? The authors must also consider how the recruitment process of the participants influence the results.

We agree that the description of the participant’s recruitment and the sampling methodology used could have been reported in a more exhaustive manner. Considerations related to participant’s enrollment, block randomization and power/sample size estimates were included in the methods and results sections of the revised manuscript (PAGE 11-12). This issue was also underlined by Reviewer 1.

We included information related to participants’ recruitment and the procedures used for allocation to experimental conditions’ order in the revised manuscript. Recruitment strategies consisted in poster advertising and personal contact between Crossfit coaches and their public. All participants expressed their interest to participate in the study, and met inclusion criteria provided in the methods with regards to background experience in weightlifting and self-myofascial release (PAGE 5). Our sample was thus representative of amateur CrossFit athletes dedicated to a regular training practice on a weekly basis (PAGE 5). Since we implemented a cross-over design, all participants underwent both experimental conditions at different time points. Hence, counterbalancing the order of the interventions was necessary to prevent order bias. This is why we implemented a block randomization with random block size selection to assign participants to one of the two possible order sequences for experimental conditions (i.e. Roller massage first, Control first). We used the *blockrand* package for R and while loop to reach the required accuracy (i.e. corresponding to the number of volunteering males and women; see also response to Reviewer 1):

Fig. 1. R script code used for block randomization and illustration of the output (replicated here to provide concrete illustration).

In keeping with Reviewer 3’s comment, this additional information was included in the methods section of the revised manuscript (PAGES 5; 7; 11).

Lines 116-122: still it’s unclear how deep was the squat. wciąż jest niejasne jak określano głębokość przysiadu. Although it was constant, it does not follow from the description of whether it is a full, maximum deep squat or to a specific angle in the knee joint.

We provided additional information regarding the general instructions for performing the maximum deep squat in this revised version (PAGE 7). Since participants had different anthropometric characteristics and different mobility and flexibility levels, they were asked to perform a deep squat up to reach their maximal knee and hip flexion amplitude. To control for correct execution of the deep squat with an empty barbell, we applied the International Powerlifting Association rules, particularly : "(…) *the lifter must bend the knees and lower the body until the top surface of the legs at the hip joint is lower than the top of the knees* "(2021). Once the maximal squat depth was reached without postural failure or compensatory movements (e.g. "butt wink"), the elastic was fixed to guarantee the reproducibility at the individual level across experimental sessions.

In the revised manuscript, additional information regarding the assessment of squat depth and the control of squat repetitions was provided (PAGE).

Line 127: please provide information about roller density

The “high” density of the roller massager was provided in the revised version (PAGE 8).

Line 128-129: how the level of pressure was determined?

The roller massager had no foam. It was made of hard plastic, hence had higher density compared to a foam roller. The pressure level was controlled using a subjective threshold, in keeping with the methodological standards adopted in previous experiments (Dębski, 2019; doi: [doi.org/10.2478/bhk-2019-0005](https://doi.org/10.2478/bhk-2019-0005)). Participants were instructed to never exceed a 7 out of 10 pain threshold when performing the self-massage routine. This was mentioned lines 144-147 of the original manuscript. While we agree that an objective measure would be preferable, the methods and devices reliable to achieve such measures remain difficult to establish at this point. Some authors used a force platform for index pressures applied using foam rolling (with the foam roller on the floor) from the weight, but could not quantify the contact surface indicative of pressures relative a given surface area (Murray, 2016; PMC5046970, Baumgart, 2019; doi : 10.3390/sports7010027). Anyhow, such approach is not applicable to self-myofascial release with a roller massager routine. Using a subjective threshold provides a reproducible methodological framework allowing for the subjective control of pressures. Also, a subjective pain threshold corresponds to actual practice contexts where athletes usually adjust their massaging actions based on their perceived sensations.

In the revised manuscript, further rationale regarding the use of a subjective pain threshold was provided, and we included these additional information (PAGE 8).

Line 130: What was rolling routine?

In order to distinguish the two conditions, passive rest and SMR, the description of the rolling routine has been moved to the experimental condition section on lines 169-177.

Line138: Typo Mars instead of March

Thanks for the careful reading. This was amended in the revised manuscript.

If familiarization session and two experimental session separated from each other by 10 consecutive days and in between days participants were instructed to avoid intensive training but otherwise we know that few consecutive days without proper stimulation could effect in lowering strength performance (e.g. Lucas Duarte Tavares, Eduardo Oliveira de Souza, Carlos Ugrinowitsch, Gilberto Candido Laurentino, Hamilton Roschel, André Yui Aihara, Fabiano Nassar Cardoso & Valmor Tricoli (2017) Effects of different strength training frequencies during reduced training period on strength and muscle cross-sectional area, European Journal of Sport Science, 17:6, 665-672, DOI: 10.1080/17461391.2017.1298673). Please comment on that.

Reviewer 3 raised here an important concern. When designing the experiment, we considered that administering shorter time delays between experimental sessions would increase risks for muscle damage impairing performances from one experimental session to another. Nonetheless, we agree that 10 days delay in the complete absence of physical practice would potentially confound the results due to a drop in performances from the one session to another. A key point that we did not explicitly mention in the original version is that athletes were instructed to avoid strenuous exercises, i.e. training at the highest intensities with the intention to outperform their CrossFit personal records, but were allowed to maintain regular practice at their maintenance levels (PAGE 5). This was expected to prevent training-related improvements from one experimental session to the other, but at the meantime to provide the necessary amounts of training to achieve maintenance. Crucially, using a cross-over was specifically expected to control for linear trends from the first to the second experimental session, i.e. such error being randomly distributed across the block randomized order for administration of experimental conditions.

These clarifications were included in the methods section of the revised manuscript (PAGE 5). We included the references suggested by Reviewer 3 to emphasize the risk for linear trends in repeated measures design measuring strength performances. We are convinced that clarifying that athletes were allowed to maintain their weightlifting practice at maintenance level is required (PAGE 7). Ultimately, addressing Reviewer 3’s concern would require independent testing of the main effect of SESSION (First, Second) on the total workload to index potential linear trends. However, considering the block randomization of experimental sessions’ order, this would leave the main analysis and results pattern unbiased.

Line 186: 15 cm above the top of the patella – why not in the widest place, it’s puzzling when you take into consideration differences between participants?

Reviewer 3 once again raised an important issue regarding the anatomical criteria used for thigh circumference. We used this anatomical criteria based on several considerations. First, thigh circumference measure are common in clinical assessments of knee functionality. It appeared that measuring thigh circumference 15 cm above the patella represents a methodological standard (e.g. Nicholas, J. J., Taylor, F. H., Buckingham, R. B., & Ottonello, D. (1976). Measurement of circumference of the knee with ordinary tape measure. *Annals of the rheumatic diseases*, *35*(3), 282-284; Soderberg, G. L., Ballantyne, B. T., & Kestel, L. L. (1996). Reliability of lower extremity girth measurements after anterior cruciate ligament reconstruction. *Physiotherapy Research International*, *1*(1), 7-16). Also, while we agree that using 15 cm above the patella exposes us with potential bias when comparing independent groups (since subjects have different femur length, for instance), participants in the present cross-over design were their own controls. Presumably, measuring thigh circumference at the thickest thigh section also exposes to bias related to anatomical differences regarding the morphology and repartition of the fat tissues, at least to a greater extent than a fixed distance from the patella. This possibly explains why 15 cm above the patella is preferred in several experiments.

We clarified the methodological approach related to thigh circumference assessment in the revised manuscript, and incorporated the above-mentioned references to strengthen the scientific background (PAGES 10; 17).

Line 302: ecological doesn’t seem to be good description for typical conditions

We agree that his terminology was misleading. We initially intended to convey the idea that this research reproduced an actual training context, hence had an applied dimension. We rephrased our statements to emphasize the practical dimension providing “ecological validity” (PAGE 16), referring to the statements of Monteiro (Monteiro, 2019; doi: [10.1016/j.jbmt.2019.01.008](https://doi.org/10.1016/j.jbmt.2019.01.008)) in the revised manuscript (PAGES 16; 20).

Lines 329-330: wrong reference style

The reference style was amended in the revised manuscript (PAGE 18).

Lines 332-335: improved muscle metabolic activity as well as blood circulation was proved in recent study in PLOS One, please see: Adamczyk J.G., Gryko K., Boguszewski D. Does the type of foam roller influence the recovery rate, thermal response and DOMS prevention? PLoS ONE 15(6): e0235195. <https://doi.org/10.1371/journal.pone.0235195>

We thank Reviewer 3 for underlining this research. It corroborates the assumption that foam rolling during recovery periods has the potential to improve the post-workout recovery. Our present design departs from this research, since foam rolling was embedded during training and not administered as part of the post-workout recovery. We nonetheless agree that this reference helps discussing the findings in relation to the state of the art.

Reviewer 3’s consideration and the reference were incorporated to the discussion section of the revised manuscript (PAGES 17-18)
