# Supplementary material for: Evaluating the effects of embedded self-massage practice on strength performance: A randomized crossover pilot trial
Source: PLoS One. 2021 Mar 2;16(3):e0248031. doi: 10.1371/journal.pone.0248031 (PMC7924734; doi:10.1371/journal.pone.0248031)
Supplement: Attachment — Submitted filename: response_to_reveiwers.docx [file pone.0248031.s008.docx]

Reviewer #2: The authors are thanked for responding to my comments. I have a few minor comments that I believe would improve clarity.

Abstract – line 22: Self-myofascial release is a misleading/erroneous term as it is questionable whether they actually “release” fascia (see, for example, Behm & Wilke, 2019, Sports Med). Please rephrase to a more appropriate term throughout the manuscript.

Replacing “foam rolling” by “self-myofascial release techniques” was a suggestion of Reviewer 3, hence our initial approach regarding the terminology. We substituted this term with “Self-administered massage interventions (…)” and “rolling” throughout the revised manuscript.

Introduction:

Line 46: Consists ‘of’

Line 73: ‘has been shown’

Line 79: The notion of ‘compounding fatigue’ which I have highlighted as problematic in my previous review remains in this section. Please revise.

We thank Reviewer 2 for the thorough reading of the revised manuscript. All typos were corrected in the present revised version. With reference to Reviewer 2’s initial comment, the inappropriate term “fatigue” was replaced by “fatiguability”, i.e. referring to the transient performance drop, as initially suggested by Reviewer 2;

Discussion:

Line 335-338: According to the definitions proposed by WHO and adopted by the American College of Sports Medicine you would not have been controlling for the ‘sex’ rather than ‘gender’ effect. Particularly as you proceed to qualify it as ‘male’ and ‘female’, which are nouns associated with sex, rather than gender.

We entirely agree with Reviewer 3. This was amended throughout the revised manuscript.

Line 365: You state “however, their [pressure] short-term effects on performance appeared here detrimental.” It is difficult to attribute this to pressure alone or rather disassociate pressure from other factors. For example, when pressure of roller massaging is controlled, the effects seem rather similar once a certain, very low baseline threshold is exceeded (Grabow et al. 2018, J Strength Cond Res).

We agree with Reviewer 3’s comment. The effects of pressure cannot be dissociated from other factors. To avoid misinterpretation, the sentence L. 365 was removed from the revised manuscript. Also, we included the reference suggested by Reviewer 3 for readers interested in the selective effects of distinct pressures intensities.
